# Monitoring Cerebral and Renal Oxygenation Status during Neonatal Digestive Surgeries Using Near Infrared Spectroscopy

**DOI:** 10.3389/fped.2017.00140

**Published:** 2017-06-14

**Authors:** Jonathan Beck, Gauthier Loron, Claire Masson, Marie-Laurence Poli-Merol, Eliane Guyot, Camille Guillot, Nathalie Bednarek, Caroline François

**Affiliations:** ^1^Neonatal and Pediatric Intensive Care Unit, CHU of Reims American Memorial Hospital, Reims, France; ^2^Department of Research and Public Health, CHU of Reims, Reims, France; ^3^Department of Pediatric Surgery, CHU of Reims Hôpital Maison Blanche, Reims, France; ^4^University of Reims Champagne Ardennes, UFR médecine, Reims, France; ^5^Department of Pediatric Anesthesia, CHU of Reims Hôpital Maison Blanche, Reims, France; ^6^Pediatric Intensive Care Unit, CHRU of Lille, Lille, France; ^7^Plastic Reconstructive Surgery and Anesthesiology, CHU of Reims Hôpital Maison Blanche, Reims, France; ^8^EA 3801 Laboratory, Champagne Ardennes University SFR CAP santé Reims-Amiens, UFR médecine, Reims, France

**Keywords:** NIRS, neonatal surgery, neonatal anesthesia, neonatal monitoring, abdominal compartment syndrome, Intraoperative monitoring, hypovolemia

## Abstract

**Background:**

Depending on the initial pathology, hypovolemia, intra-abdominal hypertension, and sepsis are often encountered in neonatal digestive surgery. Accurate newborn monitoring during and after surgery is essential to adapt resuscitation protocols. Near infrared spectroscopy (NIRS) is non-invasive and can detect hypoperfusion which indicates a low circulatory blood flow, regardless of the cause.

**Objective:**

Evaluating changes in cerebral and renal regional oxygen saturation during neonatal digestive surgeries, conducted according to normal practices, with commonly used monitoring parameters. Analyzing retrospectively the inter-relationships between NIRS values and mean arterial pressure (MAP) values as well as pre-ductal SpO_2_.

**Methods:**

Prospective, descriptive, monocentric study. All neonates referred for surgery were included. NIRS allows the measurement of cerebral and renal oxygenation fluctuations, as well as calculating difference in intraoperative and postoperative values.

**Results:**

Nineteen patients were included. Cerebral regional oxygen saturation (C rSO_2_) values were stable while renal regional oxygen saturation (R rSO_2_) values tended to decrease with time during surgery. Indeed, 72% of rSO_2_ decline episodes occurred after the first 30 min of surgery, without any significant statistical differences for the next 90 min of surgery. After surgery, the lowest average C and R rSO_2_ values were evidenced during the first 6 h, with 60% of C rSO_2_ and R rSO_2_ anomalies occurring in that time frame. There was no significant statistical difference observed in the following 18 h. There was a significant correlation between R rSO_2_ and SpO_2_ values (*p* < 0.01), but not with C rSO_2_ values. There was no correlation with the MAP either for the C rSO_2_ values or R rSO_2_ ones.

**Conclusion:**

NIRS is a promising non-invasive bedside tool to monitor cerebral and tissue perfusion, analyzing tissue microcirculation. NIRS has its interest to guide neonatal digestive surgeries (bowel manipulation, viscera reduction) and may represent an early warning for identifying patients requiring resuscitation during or after these surgeries.

## Introduction

Neonatal digestive surgery, whether to correct a malformation [gastroschisis, omphalocele, congenital diaphragmatic hernia (CDH), or esophageal atresia (EA)] or an acquired pathology such as necrotizing enterocolitis (NEC) can pose a life-threatening risk for these children. Whether a consequence of the anesthesia (vasodilatation, hypovolemia, bradycardia), or of the surgery itself (bleeding, loss of bodily fluids, hypothermia), or of related procedures to the initial pathology (reintegration of herniated viscera within the abdomen, sepsis, thoracotomy, and lateral decubitus), a monitoring of the circulatory and ventilatory statuses is mandatory in such cases (1–3).

The neonatal team needs to anticipate these adverse events. To date, only systemic measures are available such as fluid volume evaluation and blood flow status.

Fluid volume is monitored before, during, and after surgery by direct and indirect mean arterial pressure (MAP in mmHg) and heart rate (HR in beats per min) measurements. Ventilation status is documented by pre- or post-ductal pulse oximetry (SpO_2_ in%), arterial oxygen tension (PaO_2_ in mmHg), expiratory or peak inspiratory pressure (PIP in cmH_2_O), fraction of inspired oxygen (FiO_2_ in%), and measurement of end tidal CO_2_ tension (EtPCO_2_ in mmHg), *via* transcutaneous (TcPCO_2_ in mmHg) or blood (PCO_2_b in mmHg) measures (4, 5).

When abdominal compartment syndrome might be an adverse event, especially in surgery for anterior abdominal wall closure anomalies, an intra-abdominal pressure (iaP) measure could be useful. Intra-abdominal hypertension, defined by iaP > 12 mmHg (6), increases the ventilatory and circulatory risks on these fragile newborns. Even if this progressive approach, consisting in placing a silo pouch stoma to contain the viscera, it is often used when the neonate’s state is quite frail, there is no validated standard method for iaP measure in neonatal surgery (7).

Similarly, no method has been validated to assess perfusion and oxygenation quality of the different viscera during or after neonatal digestive surgeries (4, 5).

It seems essential to develop more precise regional monitoring of tissue perfusion and oxygenation, allowing early detection of adverse circulatory or ventilation events for implementing a better therapeutic response. Near infrared spectroscopy (NIRS) might have this potential in neonatal digestive surgeries.

Near infrared spectroscopy was first reported in 1977 by Jobsis as a continuous non-invasive technique measuring tissue oxygenation. Regional oxygen saturation (rSO_2_) reflects the balance between the tissues’ oxygen requirements and actual tissue oxygenation. It also reflects the microcirculation of arterial, venous, and capillary networks: 75–85% of the signal comes from venules (8–11). NIRS is interesting for detecting subclinical hypoperfusion (microcirculatory dysfunctions).

Analyzing rSO_2_ variations can assess perfusion quality in an area of interest. Many studies focused on cerebral perfusion during cardiac and vascular surgeries (12).

Little is known about the relevance of NIRS in the simultaneous monitoring of cerebral and abdominal or renal perfusion in neonatal digestive surgeries (13–16). Giliberti et al. reported and summarized the results of a study on the application of NIRS in neonatal cardiac and digestive surgeries, on its future possibilities and its relevance on perfusion and oxygenation monitoring during these surgeries and during anesthesia.

In this work, the objective was to study the NIRS renal and cerebral parameters variation during and after classic digestive neonatal surgical procedures, describing pathological rSO_2_ during and after surgeries, as well as associated events and these parameters’ behavior during fluid management (FM).

A secondary objective was to analyze correlations between NIRS parameters, MAP, and pre-ductal SpO_2_ values.

## Materials and Methods

### Subjects

Each neonate admitted in the neonatal intensive care unit (NICU), from October 2014 to November 2015, for a surgical digestive procedure (gastroschisis, omphalocele, CDH, EA, NEC, neonatal bowel obstruction, abdominal tumor) was included in the study. Neonates who died before surgery and/or had a medically managed NEC were excluded. All patients included had general anesthesia during the procedure. The study was approved by the local ethics committee of the Reims Teaching University Hospital, and all parents received full disclosure on this study and the procedure.

### Hemodynamic and Respiratory Parameters Measurement and Analysis

Mean arterial pressure, HR, and pre-ductal SpO_2_ were measured with the monitor IntelliVue MX700 (Philips Healthcare, Allmendstrasse 140, 8027 Zürich, Switzerland) associated with its multi-measures module which connected a Massimo set for SpO_2_. These non-invasive techniques adapted to age and weight. All monitoring equipment was calibrated according to manufacturer’s standards.

The hemodynamic and respiratory monitoring started at the newborn admission. During and after surgery, HR and SpO_2_ were continuously measured. During surgery, parameters’ values were recorded every 15 min by the health-care team. Postoperative, values were recorded every hour during the first 6 h, then every 3 h. Post-ductal SpO_2_ values of the lower limbs were only a recorded with pre-ductal SpO_2_ values during herniated viscera reintegration surgery (gastroschisis, omphalocele, and CDH).

Mean arterial pressure was measured and recorded every 15 min in intraoperative and every hour in postoperative during the first 6 h, then every 3 h.

During anesthesia and surgery, FiO_2_ values were systematically greater than 60% and PIP between 18 and 22 mmHg. The patients were mechanically ventilated by tracheal tube with a pressure controlled ventilation mode.

### NIRS Measurement and Analysis

Near infrared spectroscopy measurements were performed using a continuous wave system (INVOS 5100C, Somanetics, Troy, MI, USA), used the two-channel mode, with channel 1 for cerebral monitoring and channel 2 for somatic/renal monitoring. One Cerebral/Somatic oximetry Infant-Neonatal sensor (Somanetics, Troy, MI, USA), made of an emitting diode and two detectors, was used on channel 1 and one on channel 2 during this study.

The continuous wave NIRS system uses the properties of luminous absorption of photons going through the tissues by two different wavelengths (730 and 810 nm), both used in this study. Light travels from the sensor’s light emitting diode to either a proximal or distal detector, permitting separate data processing of shallow and deep optical signals. Relative changes in the absorption of near-infrared light were sampled at 10 Hz, and these values were converted to changes in the concentration of oxy-hemoglobin (HbO_2_) and deoxy-hemoglobin (HHb) based on the modified Beer–Lambert approach to obtain rSO_2_ values, according to the formula rSO_2_ = HbO_2_/(HbO_2_ + HHb).

The sensors can be placed on several parts of the body, above the organs of interest (8, 9). We used a cerebral (C) sensor which was positioned on the forehead (below the hairline) and a renal (R) sensor on the neonate’s lower back (right side) between T10 and L2, 30 min before leaving for the operating room, when the surgery was an emergency the sensors were positioned in the operating room. Cerebral and renal rSO_2_ values were continuously recorded. In the operating room, before setting up, the surgeons checked the position of the sensor and consolidated its fixation. The anesthetists made sure of uninterrupted recording.

We defined an initial C rSO_2_ and R rSO_2_ value at minute (*m*) 0 in the operating room.

These values were transformed into rSO_2_ variations in regards to initial values according to the following formula ΔrSO_2_ = ((rSO_2_measured − rSO_2_initial)/rSO_2_initial) × 100 (%). According to literature, a 20% or more decrease, compared to initial rSO_2_ values, is considered pathological (negative predictive value = 97%). NIRS standard absolute values are not referenced, due to inter and intra-individual variations, except for an inferior threshold common to all regions: a value of rSO_2_ < 50% is considered pathological (8, 9, 12, 13, 17).

All above mentioned values were recorded along with hemodynamics and respiratory parameters at the same time. On a statistical level, we studied C and R rSO_2_ variations compared to their initial values (ΔrSO_2_). At the end of the 24 h post-surgery, we also collected the mean C and R ΔrSO_2_, recorded by the device.

### Fluid Management

Fluid management and transfusions were decided prescribed at the anesthesiologist’s and neonatologist discretion during surgery and after surgery. Collected data regarding FM and transfusion were: number, type, volume (milliliters/kilogram), duration (minutes), initial ΔrSO_2_ C before and after bolus infusion (%), initial ΔrSO_2_ R before and after bolus infusion (%).

### Epidemiologic and Complications Data Collection

We collected epidemiologic and complications data for each patient: term birth (in weeks of amenorrhea), gender, birth weight (BW), APGAR at 1/5/10 min, main diagnosis, associated malformations, age at the time of surgery (in days), number of surgeries, duration of the surgery (min), resuming of urine output <6 h post-surgery, complications in the first 24 h post-surgery, duration of NICU stay (in days), total hospitalization duration (in days), and neonatal death or not.

### Statistical Analysis

Statistical analyses were performed using SAS 9.4 software. Qualitative variables were described as sample and percentages and quantitative variables as means and SD or median and Interquartile Range (IQR).

Comparative analysis to compare the average cerebral and renal ΔrSO_2_, pre and post FM were performed with a Student *t*-test for paired samples.

Comparative analyses to compare H0–H1 with H1–H2 period during surgery and H0–H6 with H6–H24 period after surgery for the cerebral and renal pathological rSO_2_ were performed with a Chi-square test.

A generalized estimation equation model with a correlation matrix for structured residues was used first to analyze independently the correlation between: (1) C and R rSO_2_ during the first day (from m0 of surgery to H24 after surgery), (2) C rSO_2_ with R rSO, (3) C and RrSO_2_ with MAP, (4) C and R rSO_2_ and pre-ductal SpO_2_, and (5) evolution of C and R rSO_2_ between operative and postoperative period. Then, we analyzed the correlation between NIRS values, MAP and pre-ductal SpO_2_ with time over the surgery and the 24 h after surgery. Results were expressed in variation (95% confidence interval). *p*-Value < 0.05 was considered statistically significant. Graphs were performed with Excel in “box plot” style with the following parameters: mean, median, minimum, maximum, quartile 25, quartile 75, decile 1, and decile 9.

## Results

### Population

Near infrared spectroscopy monitoring was performed on twenty-one patients. Two patients were excluded: reasons were death before surgery (*n* = 1) and no available perioperative data (*n* = 1). Among the 19 patients included, three patients with abdominal wall defect (two gastroschisis and one omphalocele) benefited from a silo pouch stoma allowing the progressive reintegration of the herniated viscera, as it was not feasible to perform the reintegration in one surgical step (complete closure occurred, respectively, 6, 8, and 11 days after the initial surgery). There were thirteen boys (68%) and six girls (32%). Population characteristics are listed in Table 1.

**Table 1:** Population characteristics.

Case	Pathologies	Term birth (WA + days)	APGAR 1/5/10 min	Gender	Birth weight (g)	Age at surgery (days)	Duration of surgery (min)	Length of stay in the neonatal intensive care unit (days)	Total stay duration (days)
1	Congenital diaphragmatic hernia (CDH)	40 + 5	2/x/x	F	2,414	8	120	32	37
2	Gastroschisis	36 + 3	9/10/10	M	3,235	0 (6)	100 (15)	17	49
3	CDH	40	8/9/10	M	4,714	3	180	25	27
4	CDH	39	9/9/9	M	3,190	7	90	27	30
5	Omphalocele	38	9/10/10	M	3,720	0	50	15	21
6	Omphalocele	40 + 4	10/10/10	F	3,280	1	20	3	8
7	Necrotizing enterocolitis (NEC)	38 + 1	9/10/10	M	3,100	3	30	21	28
8	Omphalocele	30 + 6	3/8/9	F	1,290	1 (9)	110 (45)	27	47
9	Occlusion	35	8/9/10	M	3,370	3	90	7	37
10	Gastroschisis	36 + 3	8/9/10	M	2,855	0	40	21	57
11	AE	40	1/5/6	M	2,900	0	230	43	84
12	NEC	25 + 3	6/8/8	M	927	38	105	116	246
13	Abdominal tumor	38 + 3	9/9/10	M	3,540	7	135	9	21
14	NEC	29 + 6	3/8/10	M	1,640	3	75	75	110
15	AE	41	9/10/10	F	2,580	0	130	8	15
16	Occlusion	28	9/8/10	M	1,275	59	60	60	93
17	Gastroschisis	37	8/9/10	F	2,945	0	20	11	36
18	CDH	40 + 2	1/6/7	M	3,150	2	165	27	43
19	Gastroschisis	32 + 6	9/10/10	F	2,500	0 (11)	20 (45)	44	x
Mean ± SD		36 ± 4.7	7 ± 3/9 ± 1/9 ± 1		2,770 ± 941	7 ± 15 (9 ± 2.5)	93 ± 59 (35 ± 17)	31 ± 28	55 ± 55
Median ± IQR		38 ± 6.1	8 ± 5/9 ± 1/10 ± 1		2,945 ± 801	2 ± 5 (9 ± 2.5)	90 ± 80 (45 ± 15)	25 ± 25	37 ± 28

*Characteristics from the second surgery are mentioned within brackets*.

*WA, weeks of amenorrhea; IQR, interquartile range*.

After the initial surgery, 16 patients urinated before H6 (84%). No death or postoperative complications were reported in this patients’ cohort.

### Evolution of the C and R rSO_2_

#### The Total Study Period (Surgery and Post-Surgery)

Mean initial C and R rSO_2_ values were, respectively, 79.11% ± 9.92 and 79.37% ± 11.31 for the first surgery; 81% ± 8.72 and 72.67% ± 11.02 for the second surgery (*n* = 3 patients).

Median variations of C and R rSO_2_, at the 24 h, were −0.6% ± 9.1 (IQR) and −5.55% ± 15.25 (IQR) for the first surgery; −4.47% ± 16.29 and −10.43% ± 4.96 for the second one. Median variations of C and R rSO_2_, at 24 h, according to the pathology are reported in Table 2.

**Table 2:** Average variation of C rSO_2_ and R rSO_2_ according to the pathology.

	Average ΔrSO_2_ C (median ± IQR)	Average ΔrSO_2_ R (median ± IQR)
Congenital diaphragmatic hernia (*n* = 4)	−2.65 ± 4.63	−13.15 ± 7.45
Gastroschisis (*n* = 4)	7.10 ± 9.8	−6.90 ± 21.10
Omphalocele (*n* = 3)	0.05 ± 8.05	−8.35 ± 5.08
Esophageal atresia (*n* = 2)	−0.30 ± 4	4.45 ± 1.85
Necrotizing enterocolitis (*n* = 3)	1.20 ± 10.80	−11.50 ± 12.20
Occlusion (*n* = 2)	−9.45 ± 4.55	−4.85 ± 4.85
Tumor (*n* = 1)	4.20	−2.70

*IQR, interquartile range*.

**Figure 1:**
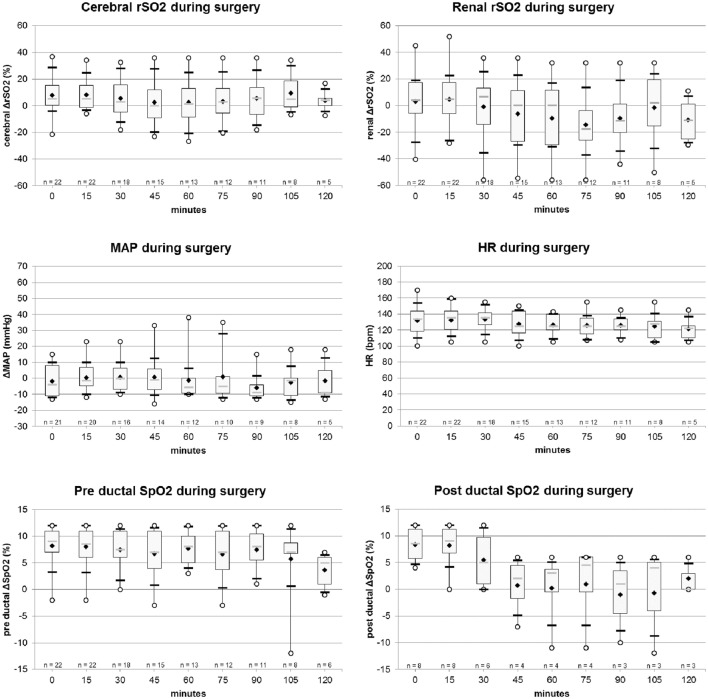
Evolution of cerebral and renal rSO_2_ (%), mean arterial pressure (MAP) (mmHg), heart rate (HR) (bpm), pre and post-ductal SpO_2_ values, during surgery. Legend: ○, minimum and maximum; ▬, first and ninth decile; gray 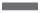, median; white ▮, quartile 25 and quartile 75; ♦, mean.

**Figure 2:**
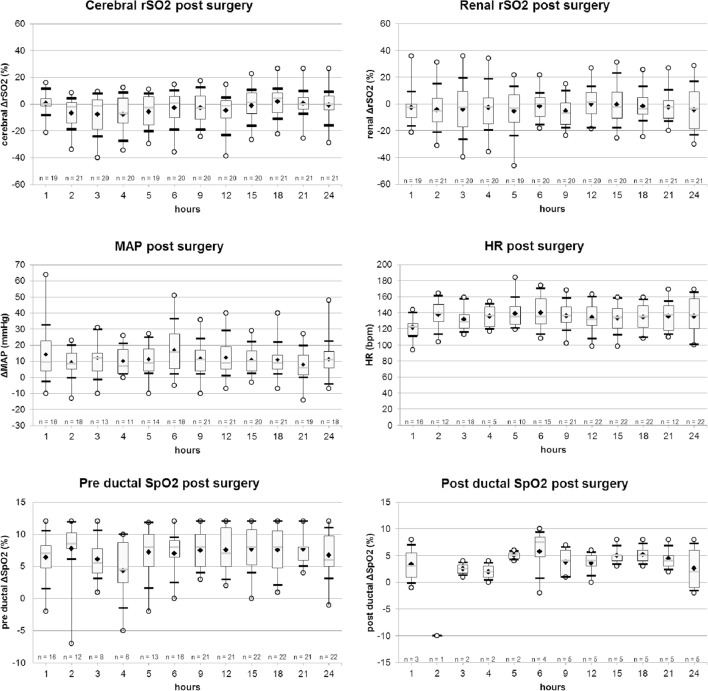
Evolution of cerebral and renal rSO_2_ (%), mean arterial pressure (MAP) (mmHg), heart rate (HR) (bpm), pre and post ductal SpO_2_ values, from H1 to H6 post-surgery. Legend: ○, minimum and maximum; ▬, first and ninth decile; gray 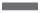, median; white ▮, quartile 25 and quartile 75; ♦, mean.

C and R rSO_2_ values, as well as hemodynamics and respiratory parameters are reported in Figures 1 and 2.

We observed a total of 90 pathological values in 12 patients; 63% (*n* = 57) R rSO_2_ and 37% (*n* = 33) C rSO_2_; 44% (*n* = 40) were reported during surgery and 55% (*n* = 50) post-surgery. The distribution of pathological values according to time is reported in Table 3 and the distribution of pathological values according to the pathology in Table 4. The pathological values and corresponding MAP, HR, and pre-ductal SpO_2_ (%) values, in regards to acute event are described in Table 5.

**Table 3:** Cerebral and renal pathological rSO_2_ values during or after surgery.

	Cerebral	Renal	Total
	
	Frequency (%)	Frequency (%)	Frequency (*n* = 90) (%)
During surgery	6 (6.7)	34 (37.8)	40 (44.5)
0–30 min	1 (1.1)	10 (11.1)	11 (12.2)
30–60 min	4 (4.5)	11 (12.2)	15 (16.7)
60–90 min	1 (1.1)	8 (8.9)	9 (10)
90–120 min	0	4 (4.5)	4 (4.5)
>120 min	0	1 (1.1)	1 (1.1)
After surgery	27 (30)	23 (25.5)	50 (55.5)
Before H6	16 (17.8)	14 (15.6)	30 (33.3)
After H6	11 (12.2)	9 (10)	20 (22.2)
Total	33 (36.7)	57 (63.3)	90 (100)

**Table 4:** Cerebral and renal pathological rSO_2_ values during or after surgery according to the pathology.

	During surgery		After surgery		Total
	Cerebral	Renal	Cerebral	Renal	Frequency (%)
Congenital diaphragmatic hernia	0	23	8	4	35 (39%)
Gastroschisis	0	1	14	5	20 (22%)
Omphalocele	1	0	0	6	7 (8%)
Esophageal atresia	2	5	0	0	7 (8%)
Necrotizing enterocolitis	1	2	1	2	6 (7%)
Occlusion	2	3	4	4	13 (14%)
Tumor	0	0	0	2	2 (2%)
Total (frequency in %)	6 (7%)	34 (38%)	27 (30%)	23 (25%)	90 (100%)

**Table 5:** Repartition of pathological rSO_2_ and corresponding mean arterial pressure (MAP) (mmHg), heart rate (HR) (bpm), and pre-ductal SpO_2_ values, in regard to acute event.

	*n*	ΔrSO_2_ (mean ± SD)	ΔMAP (mean ± SD)	HR (mean ± SD)	ΔPre-ductal SpO_2_ (mean ± SD)
Incision	4	−30.85 ± 7.91	−3.75 ± 12.82	132 ± 11.22	6.00 ± 5.89
Bowel manipulation	24	−34.69 ± 11.57	4.43 ± 16.62	134 ± 13.016	4.33 ± 4.98
Viscera reduction	4	−23.15 ± 2.27	−9.00 ± 2.83	130 ± 3.32	9.50 ± 3.00
Tracheoesophageal fistula occlusion	2	−23.80 ± 0.85	−13.00 ± 4.24	110 ± 3.54	2.00 ± 7.07
Progressive viscera reintegration	4	−24.53 ± 5.23	−0.25 ± 8.54	124 ± 8.23	9.50 ± 2.08
Abdominal wall closure	2	−27.10 ± 10.04	0.50 ± 10.61	135 ± 14.14	5.00 ± 1.41
Emesis	3	−23.03 ± 2.67	Unknown	114 ± 14.01	8 ± 0.00
Tracheal or nasotracheal tube obstruction	17	−29.41 ± 7.49	12.27 ± 13.47	134 ± 19.14	5.42 ± 4.46
Accidental ventilation arrest	7	−27.20 ± 4.79	−2 ± 13.39	145 ± 1.00	1.75 ± 2.06
Pain (EDIN >5)	15	−24.89 ± 6.18	15.75 ± 8.76	137 ± 9.45	8.44 ± 2.70
Unknown	8	−26.26 ± 6.25	11.57 ± 8.92	144 ± 15.19	8.25 ± 3.33

*EDIN, “Echelle de douleur et d’inconfort du nouveau-né” “Pain and discomfort new-born baby scaled.”*

#### Acute Events during Pathological rSO_2_

We analyzed events related to rSO_2_ pathological values. We noted that it matched with events carrying risks in the intraoperative or postoperative period (Table 5). During the intraoperative period, these events were mainly incision, bowel manipulation, viscera reduction, tracheoesophageal fistula occlusion, and abdominal wall closure. Other acute events were related to respiratory events (tracheal or nasotracheal tube obstruction and accidental ventilation arrest), progressive viscera reintegration, pain [evaluated *via* the newborn baby scaled named EDIN (18); scale as >5]. We observed emesis during pathological rSO_2_ events in the postoperative period. But in some cases (*n* = 8), no cause was identified. Interestingly, hemodynamics parameters (MAP, HR, and pre-ductal SpO_2_) did not show pathological variations.

#### Surgery

During surgery, C rSO_2_ values were stable, pathological values occurring only punctually and briefly; while average R rSO_2_ values tended to decrease gradually (Figure 1; Table 3) for the same period, without statistical significance (Table 3) There was no significant difference between m0–m30 and m30–m120 period during surgery for pathological C rSO_2_ occurrence (Chi-square = 2.669; *p* = 0.102) and R rSO_2_ (Chi-square = 1.939; *p* = 0.164). During the postoperative period, the lower mean values for C and R rSO_2_ were evidenced in the first 6 h post-surgery, gradually reversing to normal values over time. Regardless, the difference in C and R rSO_2_ parameters between H0–H6 post-surgery and those between H6– H24 was not statistically significant (C rSO_2_ Chi-square = 1.237; *p* = 0.266 and R rSO_2_ Chi-square = 1.391; *p* = 0.238).

During surgery, two patients presented both pathological C and R rS02 values: a patient with NEC (1 C rSO_2_ and 2 R sSO_2_ pathological values) and a patient with bowel obstruction (2 C rSO_2_ and 2 R sSO_2_ pathological values). Two patients presented only pathological C rSO_2_: a patient with omphalocele (1 episode) and a patient with EA (2 episodes). Seven patients presented isolated pathological R rS02: three patients with CDH (respectively, 9, 7, and 6 episodes); a patient with gastroschisis during the primary surgery for herniated viscera reintegration (with delayed final closure and silo pouch stoma; 1 episode); another patient with gastroschisis (1 episode); a patient with EA (5 episodes); and a patient with bowel obstruction (1 episode).

Hemodynamics and respiratory parameters were stable during the surgery (Figure 1).

#### The Post-Surgical Period

During the first 6 h after surgery, we observed a decrease of C rSO_2_ and a slight reduction in renal values. Moreover, most pathological values, 60% (*n* = 30/50), were observed during this period, afterward rSO_2_ tended to return progressively to its initial values.

During the post-surgical period, four patients presented both pathological C and R rSO_2_: two patients with CDH (respectively, one cerebral and one renal episode; seven cerebral episodes and two renal episodes); and two patients with bowel obstructions (respectively, two cerebral episodes and three renal episodes; three cerebral episodes, and one renal episode). Three patients presented isolated pathological C rSO_2_ episodes: a patient with NEC (1 episode), a patient with gastroschisis after the secondary abdominal closure (11 episodes, including 5 episodes of pathological values in the first 6 h post-surgery), and another patient with gastroschisis (3 episodes).

Seven patients presented isolated pathological R rSO_2_ episodes: a patient with CDH (one episode), two patients with omphalocele (three episodes each), a patient with NEC (two episodes); a patient with abdominal tumor (two episodes) and two patients with gastroschisis with delayed abdominal closure (respectively, two episodes and one episode). Decreasing NIRS values were noted after the two procedures in the first case, but only after the initial procedure in the second case.

Hemodynamics (MAP and HR) and respiratory (pre- and post-ductal SpO_2_) parameters were stable and normal (Figure 2).

### C and R rSO_2_ Correlation

The analysis of the C rSO_2_ correlation with hemodynamics parameters (MAP, SpO_2_, intraoperative or postoperative period, m0–H24 time period) was significant for the m0–H24 time period. C rSO_2_ increased by 0.32% (0.02; 0.61) (*p* < 0.05) per hour between m0 and H24, independently from all other parameters. But when we compared C rSO_2_ during the intraoperative period versus the postoperative period, we noted a significant difference of the C rSO_2_ of −10.85% (−4.28;−17.43) (*p* < 0.01). C rSO_2_ were significantly higher during intraoperative period than postoperative period. A progressive return to normal of the values is observed after the 6th hour. No correlation was found between C rSO_2_ and any of the other parameters studied.

The analysis of the correlation of R rSO_2_ with the same parameters (MAP, SpO_2_, intraoperative or postoperative period, m0–H24 time period) was also significant for the m0–H24 time period. R rSO_2_ increased by 0.71% (0.06; 1.36) (*p* < 0.05) per hour between m0 and H24. There was also a significant association between R rSO_2_ and pre-ductal SpO_2_. Renal rSO_2_ values increased by 1.50% (0.46; 2.53) (*p* < 0.01) when pre-ductal SpO_2_ values increased by 1%, independently from other parameters studied. No correlation with the other parameters studied was unveiled. Contrarily to C rSO_2_ values, there was no significant difference noted for R rSO_2_ values between the intraoperative period and postoperative period.

The C rSO_2_ with R rSO_2_ correlation analysis during the study (during and after surgery) was significant, we noted that C rSO_2_ increased by 0.22% (0.04; 0.39) (*p* < 0.05) when R rSO_2_ increased by 1%.

### Fluid Management

There were 55 FM procedures in total, 34 (62%) with albumin, 16 (29%) with crystalloids, and 5 (9%) with transfusion (4 red cell concentrates and 1 platelet transfusion). The volume of the FM was on average 10.5 ± 4.5 ml/kg and was administered in 47 ± 22 min.

No rSO_2_ data were available for nine FM. There were seven cases of pathological rSO_2_, 1 C and 6 R episodes before FM. We observed that 71% of these pathological rSO_2_ (5/7) were improved by FM: one patient with gastroschisis and one patient with omphalocele (with delayed final closure and silo pouch stoma), one CDH, one patient with bowel obstruction, and one patient abdominal tumor.

Among the non-pathological rSO_2_ patients, we noticed that only one had improved by 20% (patient with gastroschisis at the beginning of surgery, just after the transfer from NICU to operative room). The comparison of mean C and R ΔrSO_2_ values pre and post FM were, respectively, −0.39 ± 7.77 (*p* = 0.736) and 0.77 ± 18.99 (*p* = 0.786).

## Discussion

Near infrared spectroscopy monitoring was studied in multiple surgical and medical fields, for pediatric and adult patients alike (12–14, 19–22).

Based on literature data, there are no absolute values of reference for NIRS measures, regardless of the organ of interest because of inter-individual and inter-situation variations. It was previously established that a 20% decrease of the initial rSO_2_ is a sign of tissue hypoperfusion, and an absolute value of cerebral rSO_2_ < 50% underlines a strong risk of brain damage visible on imaging data with a potential risk of disability, and an absolute rSO_2_ value (cerebral or somatic) <30% is considered a vital emergency with severe visceral ischemia in progress (8, 9, 17).

Regarding neonatal digestive surgery, Conforti et al. (15) studied a series of CDH and showed that reintegration of viscera in the abdominal cavity was associated to decreased renal NIRS values. However, Westgarth-Taylor et al. used NIRS similarly to evaluate the impact of viscera reintegration on renal values. They aimed to determine if in case of CDH, oliguria was due to intra-abdominal hypertension by analyzing renal rSO_2_ during surgery. They documented that iaP increase and rSO_2_ decrease did not correlate with oliguria, which was certainly related to various factors such as frailty, FM issues, difficulty in measuring loss of fluid in a precise manner.

We observed an intraoperative reduction of renal rSO_2_ during or after herniated viscera reintegration; however, cerebral rSO_2_ values were stable in our cases, possibly explained by high FiO_2_ (>60%) and high PIP (>20 mmHg) used during the intraoperative time with sustained cerebral self-regulation. These data could possibly reflect the effectiveness of the resuscitation procedure and surgical management regarding the choice of reintegrating the herniated viscera methods (in one or two surgical times). It is well known that intra-abdominal reintegration of abdominal herniated organs induces an increase of iaP that could deteriorate abdominal tissue by compression of the inferior vena cava (15). The need for elevated PIP to fight the increased abdominal pressure in order to promote effective ventilation increases this self-sustained phenomenon.

This is, in our opinion, one of the major reasons for NIRS monitoring in these situations. It is clear that even if the use of NIRS during our study was not scientifically validated, pathological values guided our choices, mainly concerning herniated viscera reintegration in one surgery or two surgeries.

During the postoperative period, we observed that NIRS variations were the most frequent. Interestingly, the first 6 h post-surgery tended to be the most at risk in terms of pathological rSO_2_ decrease with a risk of systemic organ failure. Constant monitoring during the first 6 h post-surgery is essential. After this time, fluid balance was restored, and iaP tended to gradually return to normal levels, probably due to a decrease of the inflammation and edema, elimination of postoperative pneumoperitoneum, progressive disappearance of the ileus, and evacuation of the intra-intestinal content, as well as adaptation of the abdominal wall and skin (2, 7).

We observed a correlation between cerebral and renal rSO_2_ values and duration of care management. We also found a significant association between renal rSO_2_ values and pre-ductal SpO_2_: renal rSO_2_ values were higher when the saturation increased. NIRS being a monitoring tool for tissue oxygenation, these values were expected and only reflect the association between variations in regional saturation and global saturation (8–11).

However, we did not observe a significant association between cerebral rSO_2_ and pre-ductal SpO_2_. This absence of correlation could be explained by self-regulation of cerebral oxygenation by adaptation mechanisms to the detriment of peripheral tissue oxygenation. Regional cerebral saturation decreases when these mechanisms go into overload (15). These results promote the hypothesis that measuring both cerebral and renal rSO_2_ is highly relevant in the hemodynamic monitoring of neonatal digestive surgeries to detect central and peripheral hypoperfusion.

We did not evidence a significant association between renal and cerebral rSO_2_ and MAP. We could deduce that either rSO_2_ values were not able to predict blood pressure evolution or more probably rSO_2_ and SpO_2_ variations were occurring early on in order to implement a treatment before MAP could decrease.

We also observed that C rSO_2_ were significantly higher during the intraoperative period than the postoperative period. The high FiO_2_ (> 60%) used during the intraoperative period could be an explanation. These High FiO_2_ values could have had a major impact on the recordings in the operating room.

One other clinically relevant finding of this study was the response of NIRS to FM when rSO_2_ decreased by 20% or more. FM with adequate volumes was beneficial, particularly in case of major fluid losses frequently observed in progressive reintegration of the herniated viscera. This was also observed for red cell transfusions, probably because the increased in hemoglobin values led to a better oxygenation, and thus improved rSO_2_. These data are concordant with the literature (9, 13, 15). NIRS seems to answer independently from all other parameters used during FM (MAP, HR, SpO_2_) when measured. We believe that NIRS variations could be detected earlier than usual monitoring parameters. So NIRS anticipated changes in another parameter.

Our study had some limitations. First, the population was heterogeneous according to gestational age and BW. NIRS values in premature neonates might be different from full-term neonates, rSO_2_ values can be over or underestimated since the NIRS analytic field can vary in light of thinner skin and bone mass for identical NIRS wavelength trajectories, yet this phenomenon can be compensated by analyzing rSO_2_ variations. There could be some differences because of bilirubin levels that are often higher in premature neonates. Cerebral self-regulation differs according to birth term and postnatal age, which could impact rSO_2_ values. Second, these different pathologies are not common explaining the small sample of subjects. At last, obtaining exhaustive data was quite delicate due to recording methods (dedicated monitoring sheets). These limitations required us to carefully interpret our results, especially for FM.

However, to our knowledge, this is the first study reporting data showing the evolution of cerebral and renal regional oxygen transport during and after neonatal digestive surgeries. This type of surgery bears a major risk of homeostatic imbalance that could be life threatening to these peculiarly vulnerable patients. Using the most precise and reliable measurement of visceral oxygenation and regional microcirculation is highly relevant in these situations.

## Conclusion

Near infrared spectroscopy provides helpful information to guide the surgery (bowel manipulation, viscera reduction) and may represent an early warning for identifying patients requiring resuscitation. Monitoring during surgery and in the first 6 h post-surgery is essential, this period bearing the most risk of systemic organ failure.

## Ethics Statement

This study was carried out in accordance with the recommendations of the “Institutional Review Board of the CHU of Reims.” Our patients were too young (neonates) to give their consent. Their parents were informed of this study and the procedure and could refuse the study. Each parent of the children of this study had a standard written informed consent about the care and the use of all data concerning their child hospitalized in our university hospital, as for over patients hospitalized in our unit in our university hospital. According to our institutional review board that approved our study protocol, no specific written consent was required, just the standard written consent of our university hospital. Actually NIRS recording is considered a usual medical care, as many NICUs and operative room use it routinely in neonates. Moreover, this study was purely observational, without any decision taken or treatment introduced regarding to the NIRS information.

## Author Contributions

JB: conceptualized and designed the study, coordinated, supervised, and participated in the acquisition of data, drafted the initial manuscript, and approved the final manuscript as submitted. GL and M-LP-M: participated in the acquisition and interpretation of data, reviewed and revised the manuscript critically for important intellectual content, and approved the final manuscript as submitted. CM: carried out the initial statistical analyses, reviewed and revised the manuscript critically for important intellectual content, and approved the final manuscript as submitted. EG and CG: reviewed the conceptualization and the design of the study, participated in the acquisition and interpretation of data, reviewed and revised the manuscript critically for important intellectual content, and approved the final manuscript as submitted. NB and CF: participated to acquisition of data, reviewed and revised the manuscript critically for important intellectual content, and approved the final manuscript as submitted. All of the authors approved the final manuscript as submitted and agree to be accountable for all aspects of the work in ensuring that questions related to the accuracy or integrity of any part of the work are appropriately investigated and resolved.

## Conflict of Interest Statement

The research was conducted in the absence of any commercial or financial relationships that could be construed as a potential conflict of interest.

## Abbreviations

ACS, abdominal compartment syndrome; BW, birth weight; C, cerebral; CDH, congenital diaphragmatic hernia; CO_2_, carbon dioxide measurement; EDIN, scale of pain and discomfort of the new-born; EA, esophageal atresia; EtCO_2_, exhaled carbon dioxide measurement; FDA, food and drug administration; FiO_2_, fraction of inspirated oxygen; FM, fluid management; HHb, deoxy-hemoglobin; HbO_2_, oxy-hemoglobin; HR, heart rate; iaP, intra-abdominal pressure; IQR, interquartile ratio; IRB, institutional review board; m, min; MAP, mean arterial pressure; NEC, necrotizing enterocolitis; NICU, neonatal intensive care unit; NIRS, near infrared spectroscopy; PCO_2_b, blood carbon dioxide measurement; PIP, peak inspiratory pressure; R, renal; rSO_2_, regional tissue oxygen saturation; SaO_2_, arterial oxygen saturation; SpO_2_, oxygen saturation; TcPCO_2_, transcutaneous carbon dioxide measurement; WA, weeks of amenorrhea.
